# Between the shells: a review of acute-phase proteins in turtles

**DOI:** 10.1177/10406387261445937

**Published:** 2026-06-06

**Authors:** Shin Min Chong, Carolyn Cray, Gabriele Rossi, Shangzhe Xie, Gordon S. Howarth

**Affiliations:** Mandai Wildlife Group, Singapore; School of Animal and Veterinary Sciences, Adelaide University, Adelaide, South Australia, Australia; Miller School of Medicine, University of Miami, Miami, FL, USA; School of Veterinary Medicine, Murdoch University, Perth, Western Australia, Australia; Mandai Wildlife Group, Singapore; School of Animal and Veterinary Sciences, Adelaide University, Adelaide, South Australia, Australia; School of Animal and Veterinary Sciences, Adelaide University, Adelaide, South Australia, Australia

**Keywords:** acute-phase proteins, chelonians, inflammation, protein electrophoresis, reptiles, testudines

## Abstract

Acute-phase proteins (APPs) are used in veterinary science for diagnosing disease, identifying acute and subclinical inflammatory processes, monitoring disease progression, assessing patient response to treatment, and as a general health screening tool. In turtles, the utility of APPs, such as albumin, haptoglobin, fibrinogen, and myeloid-related protein (MRP)-126, as inflammatory markers has been investigated in a small number of studies. Serum or plasma protein electrophoresis, immunoassays, and biochemistry assays are most commonly employed to study such APPs, with mass spectrometry–based proteomics emerging as an important tool. Protein electrophoresis RIs have been established for a few turtle species. However, very few commercial assays are available for measuring specific APPs in turtles, with even fewer reported validated assays. Turtles have significant inter- and intraspecies biological variation; consequently, RIs of protein electrophoresis and specific APP assays for individual species should be established according to guidelines and referenced to determine if sex, age, reproductive status, and health status influence the results. For small populations of animals, particularly those of high conservation value, subject-based RIs are recommended if population-based RIs are not feasible. Further studies, especially biomarker identification, assay development, and validation, are required to increase the tools for disease diagnosis and monitoring of this taxon and contribute to the healthcare and conservation of many endangered turtle species.

Turtles (order *Testudines*)—reptiles with a characteristic external shell—include tortoises and terrapins. More than 50% of the 360 recognized turtle species are classified as threatened according to the International Union for Conservation of Nature Red List, which, on average, is higher than other larger classes of *Reptilia*, *Amphibia*, and *Mammalia*.^92^ Of these 360 species, 35.3% are listed as Endangered or Critically Endangered, with the main threats including habitat loss and degradation, over-harvesting of turtles and their eggs for food consumption, the global pet trade, and traditional medicinal use.^101^ However, despite their conservation status, many of these endangered animals remain poorly studied. Establishing normal health measurands and studying diseases in these animals are vital for conservation efforts.

The immune response of vertebrates is generally divided into 2 systems: the innate immune system and the adaptive immune system.^120^ Both systems include cellular and humoral components and are triggered by different endogenous and exogenous damage-associated molecular patterns (**DAMPs**) and pathogen-associated molecule patterns (**PAMPs**).^70^ Microbial infection and tissue damage, such as trauma, neoplasia, and metabolic derangements, can trigger inflammation, resulting in clinical or subclinical inflammation. Innate effector cells, such as phagocytes, release intercellular signaling molecules, including chemokines, interleukins, growth factors, interferons, and tumor necrosis factor (TNF); these proceed to activate the innate immune response.^36,70^ Upon activation, the innate immune response amplifies inflammation and initiates tissue repair, ultimately aiming to restore homeostasis.^70^

The innate immune system is nonspecific, is the first line of defense in most vertebrates, and is considered the primary defense mechanism in reptiles.^33,119^ When local inflammation is severe and spreads to other organs, biochemical signaling is amplified by increased pro-inflammatory cytokine production, such as IL1, IL6, and TNF, and a systemic acute-phase response (**APR**) is triggered. Homologues of these cytokines with similar functions to mammals have been detected in reptiles, including IL1β, IL6, and a TNF-homologue expression.^98^ Innate effector cells, including granulocytes and phagocytes, are recruited to the site of inflammation and release more pro-inflammatory cytokines and chemokines that further mediate the inflammation. These cytokines act primarily on the liver to initiate the production of acute-phase proteins (**APPs**).^36^ APPs are highly conserved and a complex component of the innate immunity and inflammatory response in vertebrates. This response includes behavioral, biochemical, and nutritional changes. In mammals, clinically obvious manifestations include fever, lethargy, and depression. Biologically, leukocytosis occurs, and APP concentrations increase or decrease in the blood, bodily fluids, or tissues, acting to amplify or inhibit the inflammatory processes locally or systemically to restore homeostasis.^13,18,91^ Given their high sensitivity to inflammatory processes, APPs have been used as quantitative markers in mammals and non-mammals for disease diagnosis, prognosis, monitoring disease progress, response to therapy, and as a general health screening tool (**Table 1**).^52,68,88,99,100,103^

**Table 1.** General classification of acute-phase proteins.^34^

Class/Subclass	Change in concentration	Time of change
Positive		
Major	Increase >100-fold	24–48 h
Moderate	Increase >5–10-fold	>72 h
Minor	Increase by 50–100%	Gradual, wk
Negative	Decrease >25%	24–48 h

In mammals, APPs are produced predominantly by the liver, although some are synthesized extrahepatically.^34,36,97^ Extrahepatic sites of synthesis include lung, adipose tissue, intestine, mammary gland, leukocytes, endothelial cells, and skeletal muscle.^34,70^ In chickens, APPs are expressed by the cecal tonsils, lung, spleen, pericardial adipose tissue, gastrointestinal mucosa, and skeletal muscle.^73,95^ In turtles, APPs have been reported to be produced by the liver, kidney, and spleen.^47,117,118^ Concentrations of APPs in the blood can be measured by biochemical assays, protein electrophoresis, specific APP assays, and proteomics.

Research on reptilian immunity and APPs is limited and influenced by numerous factors, including differences in inflammatory responses and mammalian APP homologues, biological variation, internal physiology, concurrent immunologic responses, and external factors such as season or weather.^37,62^ Studies have shown various influences of sex and age on immunity, as well as the effect of stress or disease on innate immunity.^1,6,15,33,42^ The effect of reproduction on turtle immune function remains unclear, with experimental challenges in turtles yielding inconsistent findings about inflammatory responses.^37^ Inflammation in reptiles is not always correlated with conventional clinical findings, such as leukocytosis, neutrophilia, or toxic changes; however, behavioral fever and other signs, such as anorexia, may manifest.^3,32,37,94^ Serum protein electrophoresis or APP assays are appealing in reptilian medicine as a tool complementary to conventional blood tests, given their sensitivity to inflammatory processes.^4,17,59,94^ Many studies have reported protein electrophoresis RIs in turtles, although the influence of biological and external factors, such as sex, reproductive status, presence of disease, and seasons, on protein electrophoresis and APP assays has not been reported consistently. As an overarching challenge, very few commercial assays exist for measuring APP in turtles, with only one ELISA kit for turtle myeloid-related protein (**MRP**)**-126** (Life Diagnostics) available.

Here, we review the modern knowledge base of APPs and their clinical application in turtles. For this narrative review, we searched ProQuest and Scopus with the terms “acute-phase”, “protein electrophoresis”, “turtle”, “tortoise”, “reptile”, “health assessment”, “innate immunity”, “plasma proteome”, and “inflammation” for the period 2003–2025. A graduate research thesis was referred from colleagues. Abstracts were reviewed to identify relevant articles, and we excluded articles that did not provide information on turtle health or immunity, inflammatory or disease response, or protein electrophoresis.

## Measurement of acute-phase proteins

### Biochemical assays

Some APPs can be detected by automated biochemical analyzers based on their chemical, physical, or biological activity. Albumin is routinely measured by automated analyzers using the bromocresol green (BCG) dye method. However, this method is unsuitable for reptiles and avian species because BCG binds to globulins.^21,76^ Albumin concentrations measured by the BCG method and by protein electrophoresis are weakly to moderately correlated in healthy animals but can differ significantly in diseased animals.^17,66,76^ Therefore, albumin measurements in reptiles should not employ the BCG method. Hemoglobin (**Hb**)-binding protein (**HBP**)—the analog of haptoglobin (**Hp**) in mammals—has been measured via colorimetric assays in box turtles (*Terrapene* spp.) and loggerhead sea turtles (*Caretta caretta*).^2,30,42^ Further research is required in other turtles and reptiles to determine if the Hp assays are suitable for use.

### Fibrinogen measurement

Fibrinogen in plasma can be measured by gravimetric, heat precipitation, or Clauss methods. The Clauss method is utilized most commonly by laboratories to determine the fibrinogen quantity. It is a quantitative assay that defines the ability of fibrinogen to form a fibrin clot after being exposed to thrombin.^102^ However, the Clauss method can be unreliable given the lack of a species-specific fibrinogen standard curve during validation, and has been reported to provide inconsistent results in red-eared sliders (*Trachemys scripta elegans*).^75^ The heat precipitation method is a crude method of measuring fibrinogen and may not detect inflammation or decreased fibrinogen concentrations in animals. Fibrinogen has been determined by the heat precipitation or Clauss methods in gopher tortoises (*Gopherus* sp.) and ornate box turtles.^82,94^

### Erythrocyte sedimentation rate

The erythrocyte sedimentation rate (**ESR**) is used in human, equine, and marine mammal medicine as an inflammation marker.^43,61^ Inflammation elevates fibrinogen concentrations, causing RBCs to form rouleaux, which sediment more rapidly under gravity than individual RBCs. ESR is determined using the Wintrobe method or manually with a microhematocrit tube. The Wintrobe method measures the distance RBCs settle to the bottom of an elongated 100-mm tube under the influence of gravity after 1 h.^105^ RIs for ESR have been established in healthy gopher tortoises, eastern box turtles, and ornate box turtles, with elevated rates observed in unhealthy box turtles.^2,94^

### Agarose gel electrophoresis

**Figure 1.**
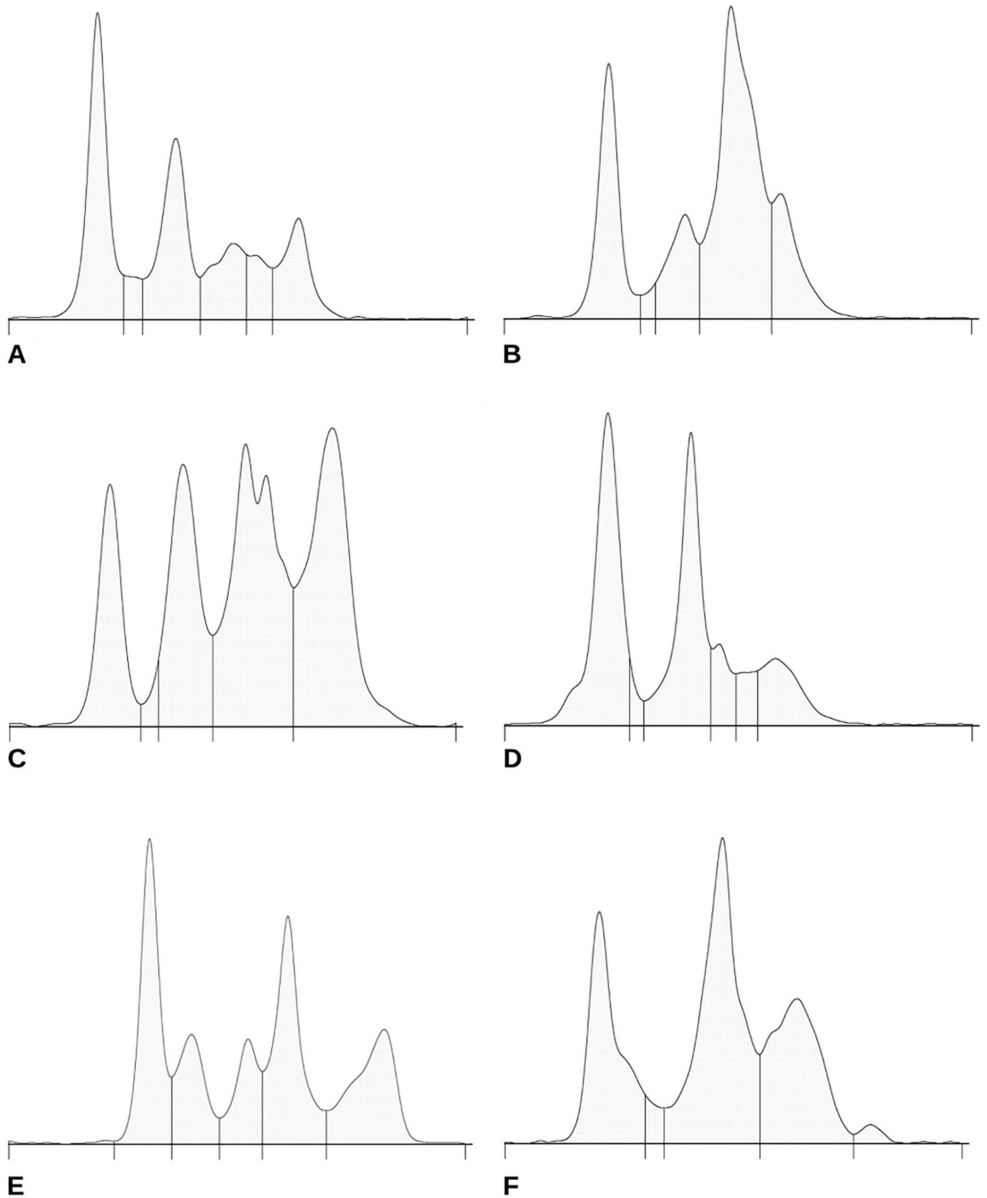
Serum protein electrophoresis electrophoretograms of healthy male turtles with wide diversity among species: **A**. *Mauremys annamensis*; **B**. *Batagur borneoensis*; **C**. *Aldabrachelys gigantea*; **D**. *Astrochelys radiata*; **E**. *Carettochelys insculpta*; **F**. *Heosemys grandis*. For A and D, hash marks indicate (from left to right) fractions of albumin and α1-, α2-, β1-, β2-, and γ-globulins. Fractions for B, C, E, and F include albumin and α1-, α2-, β-, and γ-globulins.

Protein electrophoresis (**PEP**) of serum or plasma proteins, utilizing agarose gel electrophoresis (**AGE**), is a well-established analytical technique. By applying an electric current to serum or plasma in an alkaline buffer, proteins are separated based on size, charge, and pH. The primary fractions identified through PEP include albumin, α1-, α2-, β1-, β2-, and γ-globulins.^34^ Following electrophoresis, gels are stained and scanned with a densitometer to quantify the protein concentration in each fraction, producing an electrophoretogram (**Fig. 1**). In mammals, the α-globulin fraction includes APPs such as α1-acid glycoprotein, α1-antitrypsin, α1-lipoprotein, ceruloplasmin, Hp, α2-macroglobulin, and serum amyloid A (**SAA**).^5,22^ The β-globulin fraction includes fibrinogen (plasma only), β2-lipoprotein, transferrin, ferritin, and complement components.^5^ The γ-fraction consists predominantly of immunoglobulins produced by B lymphocytes, which are integral to the humoral component of the adaptive immune system.^5,55^ PEP is invaluable for detecting abnormal protein concentrations, with AGE being the most common method for establishing RIs and investigating normal and abnormal blood protein concentrations in turtle studies.^17,19,20,58,66^ In light of the invalidity of BCG methods in the quantification of albumin, PEP is considered the gold standard in reptilian medicine to accurately measure albumin and globulin concentrations.^21,66,76^ Although PEP has high sensitivity in detecting disease or inflammation, a finding similar in mammals, it suffers from low specificity and should be used in parallel with other testing modalities.^17,32,35,91,97^

### Capillary zone electrophoresis

Capillary zone electrophoresis (**CZE**) is a technique for the electrophoretic separation of proteins in a liquid phase, utilizing a narrow-bore capillary column. The application of high voltage across the column generates an electro-osmotic flow, propelling proteins rapidly from the anode to cathode.^51^ Users have reported that, compared with AGE, CZE is rapid, offers improved resolution by generating a greater number of peaks in the electrophoretogram, is user-friendly with full automation, has lower intra-assay CV variation, and generates reproducible analyses for individual samples.^23,24,51^ A study comparing AGE and CZE methods on green turtle (*Chelonia mydas*) plasma samples found that CZE had higher precision, ease of use, and superior resolution of additional protein fractions compared with AGE.^106^

However, CZE requires specialized equipment that may not be readily accessible to diagnostic laboratories because of its cost and availability. In veterinary medicine, the presence of unknown subpeaks (resulting from increased resolution) may also pose challenges for operators. Such unknown peaks are not recommended as biomarkers of disease in mammals and non-mammals until their composition is identified, a process that is difficult and requires advanced research methods, such as immunoelectrophoresis or proteomics.^51,69,106^ Subpeaks identified in mouse samples by CZE were extrapolated from human studies; Hp was identified in the α2 fraction of cheetah (*Acinonyx jubatus*) samples by the addition of Hb-lysate for analysis.^23,28^ Further studies are required to establish the clinical usefulness and relevance of this modality in the veterinary setting. In Asian elephants (*Elephas maximus*), CZE identified a higher α2 peak in an unwell elephant compared with the AGE method. CZE fractions were significantly different between healthy and critically ill giant pandas (*Ailuropoda melanoleuca*).^80,83^ However, no significant changes in CZE fractions were found in clinically abnormal Aldabra giant tortoises (*Aldabrachelys gigantea*).^29^

### Immunoassays

Some APP proteins, such as Hp or MRP-126, may be detected using monoclonal or polyclonal antibodies to obtain qualitative or quantitative data. ELISA is commonly used in the laboratory setting to detect specific proteins and hormones, immunoglobulins, and pathogen antigens. However, separate kits are required for each protein, which may be costly and time-consuming. Each protein test also requires validation for each species to be deemed accurate and precise.^57^ Ideally, as additional biomarkers are identified, automated assays should be developed to facilitate ease of use, testing on demand, and improve result reliability, rather than employing batch analysis requiring labor-intensive ELISA.

### Proteomics

Proteomics involves the large-scale study of protein constituents in cells, tissues, organisms, or body fluids to characterize the structure and function of the protein identified.^49^ In particular, accessibility to mass-spectrometry (MS)-based techniques has advanced significantly in recent years. Such techniques can provide highly sensitive and specific identification of hundreds to thousands of proteins in blood, bodily fluids, or tissues, aiding in the understanding and diagnosis of various human diseases such as cancer, cardiovascular disease, inflammation, and infectious diseases.^14,48^ Proteomics has been used to identify APPs as potential biomarkers in mammals and non-mammals, particularly for detecting pathologic changes in the innate immune response, given their sensitivity and reliability.^49,71,81^ Additionally, proteomics can be used to monitor wildlife populations and assess the impact of anthropogenic activities or toxins on animal health. For instance, in an analysis of the blood proteome in various green turtle populations exposed to different environmental chemicals, 11 of 17 of the most highly dysregulated proteins were APPs, including α2-macroglobulin, ceruloplasmin, complement c4, complement factor d, fibrinogen α-chain, Hp, albumin, and α1-anti-trypsin.^15^

A study on plasma proteome changes in moribund and recovered green turtles found that 231 of 488 (47.3%) identified proteins had significant changes in abundance between the 2 health states, with 34 proteins having a ±5.1-fold change.^71^ Moribund turtles had greater numbers of proteins that aligned with gene ontology terms associated with complement activity, coagulation pathways, APR, adaptive immune responses, and platelet degranulation. Recovered turtles had higher numbers of proteins associated with metabolic processes, such as cellular protein, response to nutrient, negative regulation of apoptotic processes, and retinol metabolic processes. In another study of rehabilitated diseased loggerhead sea turtles, 18 of 913 plasma proteins identified by proteomic analysis differed significantly among age groups, and statistically significant differences in abundance of 20 proteins were present in animals between before and after successful rehabilitation.^54^ Of these proteins, 17 of 20 included APPs involved in regulation of the complement cascade, activation of C3 and C5, the innate immune system, and heme scavenging.^54^

As proteomics becomes more affordable and accessible, its potential for investigating and studying wildlife health will expand significantly, benefiting conservation efforts. However, a major obstacle is the unavailability of a comprehensive turtle species-specific database of genomic or protein sequences as reference for proteomic studies, as well as bioinformatics expertise for interpretation.

### Transcriptomics

By measuring mRNA concentrations with sequencing technology, gene transcription studies enable the identification and quantification of specific genes responding to environmental stressors.^32^ This detection can precede changes in hematology, biochemistry, or clinical signs. In turtle research, this technology has elucidated systemic health effects of dietary changes, malnutrition, stress, infection, and environmental changes.^10,32,47,64,110,117,118^ In Agassiz’s desert tortoises (*Gopherus agassizii*), significantly elevated blood transcription levels of genes, such as superoxide dismutase (defends against cellular and oxidative stress), myeloid differentiation primary response 88 (defends against microbial pathogens), cathepsin L (involved in protein synthesis), and leptin (associated with energy balance), were observed in clinically abnormal animals. These changes indicate roles in microbial defense, cellular and oxidative stress responses, protein synthesis, and metabolism.^10,32^ In Chinese soft-shelled turtles (*Pelodiscus sinensis*) experimentally infected with *Aeromonas hydrophilia*, the resistant group had higher expression of genes related to cell cycle signaling and pathogen defense, particularly CD3 and CD45. These cell markers, for T lymphocytes and leukocytes respectively, suggest a role for T-lymphocyte activation in resistance.^47^ Additionally, turtles experimentally infected with *Edwardsiella tarda* had upregulation of genes involved in complement and coagulation cascades and phagosome activities in the liver, highlighting their defensive roles.^64^ However, a study found that clinical health metrics did not always align with gene transcription profiles in healthy wild versus diseased captive individuals.^10^ The application of transcriptomics in turtle health research is nascent, necessitating further studies to assess its clinical utility.

## Assay development and validation

Tremendous advancement has occurred in advanced proteomics for protein identification and quantification as potential medical biomarkers in recent years, with MS-based protocols leading in this field.^48^ Before MS techniques were more readily available and accessible, veterinary APP research and assay development were traditionally performed via immunoassays or gel-based proteomics, extrapolated from human APP knowledge and applied to veterinary domesticated mammals because of a presumed high degree of conservation of APP in mammals.^35,57,67^ Subsequent species-specific research allowed identification of the main APPs in laboratory and domestic mammals, such as non-human primates, mice, dogs, horses, pigs, and cattle.^18,57^ Such species-specific research is not feasible for wildlife species. Therefore, the use of APP testing in wildlife species relies on assays developed for domestic species, extrapolated to the closest genus, family, or order.^9,43,53,60,84^ Although identification of potential biomarkers in wildlife species may now be possible with MS proteomic techniques, assay development remains a significant challenge for non-mammals and especially for turtles, considering the sheer diversity of species, evolutionary differences, and lack of commercial species-specific reagents and control materials. In turtles, MRP-126 was identified in green turtle plasma by proteomics as a potential inflammatory biomarker and, subsequently, was developed into an ELISA (Life Diagnostics).^71^

After an assay is developed to detect and quantify an APP, it must be evaluated in a robust and stepwise manner to ensure it is fit for purpose. Assay validation determines the suitability of a test method for its intended use.^39^ A proposed 4-step validation process^57^ includes:

1. Analytical performance: evaluates bias, detection limit, imprecision, inaccuracy, linearity, and reportable range.
2. Sample comparison: compares samples from healthy and diseased individuals to identify significant differences or overlaps.
3. Diagnostic performance: assesses sensitivity and specificity in a clinical setting against a “gold-standard.”
4. Usefulness assessment: determines the assay utility for the laboratory, community, or industry.

Assay validation ensures reproducible results across different users or laboratories, particularly for new test methods. The American Society for Veterinary Clinical Pathology (ASVCP) offers guidelines for assay validation.^8^ Validating assays for wildlife samples presents challenges because of logistical issues, unclear health statuses, and limited validated “gold standard” tests. Incomplete validations for APPs in non-domestic mammals have been reported, with many focusing only on imprecision and linearity.^53^ A few studies have partially validated assays for APPs in turtles,^2,29,30,42^ with most lacking complete validation. Of 9 reviewed studies (**Table 2**), 3 performed analytical validation on HBP,^2,30,42^ 2 on fibrinogen,^75,94^ and 2 on MRP-126.^29,72^ None of the assays had completed the 4-step validation process.

**Table 2.** Validation of acute-phase proteins in turtles.

Species	Analyte	Validation performed	Ref.
Box turtle (*Terrapene* spp.)	HBP	Yes	^2^
Eastern box turtle (*Terrapene carolina carolina*)	HBP	Yes	^42^
Loggerhead turtle (*Caretta caretta*)	HBP	Yes	^30^
Ornate box turtle (*Terrapene ornata ornata*)	Fibrinogen	No	^82^
Gopher tortoise (*Gopherus polyphemus*)	Fibrinogen	Yes	^94^
	Fibrinogen	No	^93^
Red-eared slider (*Trachemys scripta elegans*)	Fibrinogen	Yes	^75^
Aldabra giant tortoise (*Aldabrachelys gigantea*)	MRP-126	Yes	^29^
Green turtle (*Chelonia mydas*)	MRP-126	Yes	^72^
Loggerhead turtle (*Caretta caretta*)	MRP-126	Yes	^72^

HBP = hemoglobin binding protein; MRP-126 = myeloid-related protein 126.

## Reference intervals

RIs, based on population values encompassing 95% of a healthy reference population, are crucial for clinical decision-making.^46^ Clinicians use these intervals to interpret test results for diagnosing disease and establishing differential diagnoses. However, establishing RIs in wildlife is challenging given unknown health status, small sample volumes, field conditions, and lack of validated assays. In turtles, this is further complicated by significant biological variation among species, within species, and individuals. Given this limitation, *subject-based RIs* may be preferable for detecting and monitoring disease in individuals, benefiting small groups in zoologic settings or endangered species.^46^

A *subject-based RI* is a personalized range of laboratory values derived from an individual animal’s own baseline healthy data collected serially over a period of time, and is also referred to as the reference change value (**RCV**).^46^ Establishing subject-based RIs requires careful selection of healthy reference subjects, control of pre-analytical variation, identification and removal of outliers, and statistical analysis of data distribution before interval generation.^46^

The ASVCP has published guidelines for the establishment of de novo RIs for veterinary specimens,^45^ with 10 of 25 studies in our review reporting protein electrophoresis RIs adhering to these standards. Various turtle species have reported PEP RIs (**Table 3**). Details of protein values in various turtle species affected by biological or external factors have been reported ( **Suppl. Table 1** ).

**Table 3.** Protein electrophoresis RIs of selected turtle species. All samples are plasma unless indicated as serum (*).

Species	*Terrapene carolina carolina* ^42^		*Terrapene ornata ornata* ^1^		*Geochelone radiata* ^115^		*Geochelone radiata* ^115^		
Geographic location	Tennessee and Illinois, USA		Illinois, USA		Georgia, USA		Georgia, USA		
Sex	Male and female		Male and female		Male		Female		
*n*	320		102		10		8		
Season	Spring, summer, autumn		Spring		Winter	Summer	Winter	Summer	
Method	AGE		AGE		AGE		AGE		
Kit	Split Beta, SPIFE 3000; Helena Laboratories		Split Beta, SPIFE 3000; Helena Laboratories		Paragon Protein SPE-II electrophoresis kit; Beckman-Coulter		Paragon Protein SPE-II electrophoresis kit; Beckman-Coulter		
TP, g/L	32.8–39.0‡		23.2–89.4‡		51 ± 10§	53 ± 4§	44 ± 10§	54 ± 1§	
Pre-albumin, g/L	0.0–0.02‡		0–2.3‡		3 ± 1§	4 ± 1§	3 ± 1§	3 ± 1§	
Albumin, g/L	7.1–8.5†		6.2–23.4‡		16 ± 2§	17 ± 2§	14 ± 4§	16 ± 5§	
Globulins									
⍺1, g/L	2.5–3.0‡		2.3–8.2‡		2 ± 0.2§	1 ± 0.2§	2 ± 0.6§	3 ± 1§	
⍺2, g/L	7.6 – 9.2‡		5.6–27.8‡		15 ± 2§	15 ± 2§	10 ± 3§	14 ± 4§	
Total β, g/L	12.7–15.5‡		6.1–25.3‡		9 ± 1§	9 ± 1§	10 ± 2§	12 ± 2§	
γ, g/L	2.7–3.2‡		1.7–7.4‡		6 ± 1§	6 ± 1§	6 ± 2§	6 ± 1§	
A:G ratio	0.27–0.31†		0.27–0.54‡		6 ± 1§	7 ± 1§	6 ± 1§	5 ± 1§	
Species	*Emydoidea blandingii*^6^¤		*Trachemys scripta* ^50^		*Chelodina mccordi* (*)^17^¤		*Dermochelys coriacea* (*)^87^¤		*Dermochelys coriacea* ^26^
Geographic location	Illinois, USA		Barcelona, Spain		Singapore		Virgin Islands, USA		Republic of Gabon
Sex	Male and female		Female		Male and female		Female, nesting		Female, nesting
*n*	193–196		22		22		129		10–12
Season	Summer		Not stated		March		Apr–July		Jan, Feb
Method	AGE		AGE		AGE		AGE		AGE
Kit	Split Beta, SPIFE 3000; Helena Laboratories		Hydragel Protein (E) Hydrasys 2; Sebia		Hydragel Protein (E) Hydrasys 2; Sebia		Split Beta, SPIFE 3000; Helena Laboratories		Paragon Protein SPEP-II electrophoresis kit; Beckman-Coulter
TP, g/L	15.7–52.3‡		33.1 ± 8.3§		37.8–65.1¦		37.9–63.1¦		40 ± 10§
Pre-albumin, g/L	0.00–2.00‡		—		4.06–15.65¦		0.0–0.0¦		0 ± 0§
Albumin, g/L	2.50–13.40‡		10.3 ± 4.0§		0.49–10.44¦		14.4–22.7¦		18.1 ± 3.7§
Globulins									
⍺1, g/L	0.80–5.70‡		—		5.81–12.82¦		5.4–13.9¦		1.6 ± 0.7§
⍺2, g/L	2.10–10.00‡		—		4.12–7.82¦		2.2–14.4¦		8.2 ± 2.0§
Total ⍺, g/L	—		8.5 ±. 2.3§		—		10.3–23.3¦		—
Total β, g/L	4.00–25.70‡		13.0 ± 3.5§		7.44–16.11¦		2.5–9.7¦		8.0 ± 1.1§
γ, g/L	1.70–14.10‡		1.6 ± 1.2§		3.49–9.71¦		5.2–10.6¦		8.1 ± 2.1§
A:G ratio	0.17–0.43¦		0.50 ± 0.1§		0.21–0.88¦		0.47–0.76¦		—
Species	*Testudo hermanni* (*)^38^¤		*Gopherus polyphemus*^94^¤		***Chrysemys picta*** ^109^		*Clemmys guttata*^12^¤		*Aldabrachelys gigantea*^29^¤
Geographic location	Italy		Florida, USA		Indiana, USA		Texas, USA		USA, not specified
Sex	Male and female		Male and female		Male and female		Male and female		Male and female
*n*	22		23		37		32		27
Season	Spring		Spring, summer, autumn		Spring		Dec–Jan		—
Method	CAE		AGE		AGE		CZE		CZE
Kit	Auto Phor 400, Bio Group Medical System, AdaLya 24; Seleo Engineering		Split Beta, SPIFE 3000; Helena Laboratories		Not stated		Capillarys 2 Flex piercing; Sebia		Capillarys 2 Flex piercing; Sebia
TP, g/L	14.4–29.7¦		15.0–53.8¦		35–40†		19–66¦		16–67¦
Pre-albumin, g/L	—		1.8–8.6¦		1.1–1.5†		0.0–3.4¦		PA1: 0.00–0.7¦PA2: 0.00–8.3¦
Albumin, g/L	8.7–16.3¦		15.0–9.5¦		5.9–6.9†		2.7–13¦		2.3–7.9¦
Globulins									
⍺1, g/L	—		0.6–2.5¦		1.4–1.6†		2.3–6.5¦		1.8–21.9¦
⍺2, g/L	—		1.8–9.6¦		6.5–7.6†		5.1–21.3¦		0–13.2¦
Total ⍺, g/L	0.9–5.5¦		—		—		—		—
β1, g/L	—		—		—		0.2–5.5¦		—
β2, g/L	—		—		—		0.8–6.4¦		—
Total β, g/L	0–3.6¦		3.2–21.2¦		8.8–10.8†		2.3–10.6¦		2.2–13.4¦
γ, g/L	0.1–1.2¦		2.3–7.4¦		10.2–11.7†		19–15.2¦		1.5–11¦
A:G ratio	0.61–0.87¦		0.29–0.73¦		0.25–0.28†		0.18–0.39¦		0.12–0.34¦
Species	*Ocadia sinensis* ^16^		*Ocadia sinensis* ^16^		*Mauremys mutica* ^16^		*Mauremys mutica* ^16^		
Geographic location	Taiwan		Taiwan		Taiwan		Taiwan		
Sex	Male		Female		Male		Female		
*n*	15	15	15	15	11	11	24	24	
Season	Summer	Winter	Summer	Winter	Summer	Winter	Summer	Winter	
Method	CAE		CAE		CAE		CAE		
Kit	TITAN III cellulose acetate plate, SPIFE 3000; Helena Laboratories		TITAN III cellulose acetate plate, SPIFE 3000; Helena Laboratories		TITAN III cellulose acetate plate, SPIFE 3000; Helena Laboratories		TITAN III cellulose acetate plate, SPIFE 3000; Helena Laboratories		
TP, g/L	31–62#	27–63#	36–74#	23–59#	30–55#	30–46#	17–41#	30–42#	
Albumin, g/L	6.0–13.9#	5.8–12.4#	8.3–13.5#	4.4–11.9#	4.1–15.4#	8.6–12.9#	3.0–9.9#	7.9–12.7#	
Globulins									
⍺1, g/L	—	—	—	—	13–30#	0.8–2.7#	1.4–5.2#	0.6–4.0#	
⍺2, g/L	—	—	—	—	3.7–9.8#	4.7–11#	2.7–8.0#	4.3–11.6#	
Total ⍺, g/L	11.2–14.5#	6.4–15.5#	10–20.2#	6.2–19.8#	—	—	—	—	
Total β, g/L	11.2–21.6#	8.1–17.7#	12.6–33.2#	8–16#	7.5–14.1#	9.8–15.1#	6.1–13.8#	8.5–15.1#	
γ, g/L	3.5–13.3#	4.3–14.9#	6.1–15.0#	4.4–11.1#	4.1–16.8#	4.3–8.6#	3.7–11.6#	2.3–7.0#	
A:G ratio	0.2–0.52#	0.18–0.48#	0.17–0.46#	0.24–0.39#	0.25–0.42#	0.32–0.48#	0.21–0.36#	0.31–0.55#	
Species	*Caretta carretta* ^79^		*Caretta carretta* ^27^		*Chelonia mydas* ^79^		*Chelonia mydas* ^40^		*Lepidochelys kempii*^90^¤
Geographic location	Florida, USA		Georgia, USA		Florida, USA		Queensland, Australia		Georgia, USA
Sex	Male and female		Female, nesting		Male and female juveniles		Male and females, all ages		Male and female
*n*	437		24		152		16-55		34
Season	All seasons		Summer		All seasons		All seasons		Summer
Method	AGE		AGE		AGE		AGE		AGE
Kit	Paragon Protein SPE-II electrophoresis kit; Beckman-Coulter		Paragon Protein SPE-II electrophoresis kit; Beckman-Coulter		Paragon Protein SPE-II electrophoresis kit; Beckman-Coulter		TITAN GEL serum protein system kits; Helena Laboratories		SPIFE 3000; Helena Laboratories
TP, g/L	22–52†		52 ± 5§		20–54†		20.8–62.1¦		26–50¦
Pre-albumin, g/L	—		0–0.11‡		—		—		0.6–5.7¦
Albumin, g/L	4.8–14.8†		11.5 ± 2.9§		7.5–21.3†		10.78–23.29¦		4.5–11.0¦
Globulins									
⍺1, g/L	—		1.5 ± 0.6§		—		—		1.2–5.5¦
⍺2, g/L	—		1.5–6.5‡		—		—		2.1–6.4¦
Total ⍺, g/L	2.3–8.5†		—		2.9–8.2†		3.97–11.05¦		—
Total β, g/L	4.3–13.2†		17.1 ± 5.2§		3.0–10.5†		1.38–10.98¦		5.5–14.8¦
γ, g/L	4.8–23.8†		11.8 ± 3.7§		3.3–19.2†		2.8–17.9¦		5.4–16.1¦
A:G ratio	0.03–0.71†		—		0.47–1.00†		—		0.22–0.51¦

AGE = agarose gel electrophoresis; CAE = cellulose acetate electrophoresis; CZE = capillary zone electrophoresis; dash (—) = not measured.

† 95% CIs; ‡ 10–90th percentile; § $\bar{x}$ ± SD; ¦ 90% CIs; # minimum-to-maximum values; published following American Society for Veterinary Clinical Pathology guidelines.

## Clinical application of acute-phase proteins in turtles

Although studies of APP application in non-domesticated animals have increased in the last 20 y, research remains limited for turtles.^53^ One of the main obstacles has been the lack of RIs for healthy populations and the large biological variation among species and within individuals. Age, sex, reproductive status, presence of disease, health status, size, or mass can affect blood protein concentrations.

Higher concentrations of plasma total protein were reported in female eastern box turtles (*Terrapene carolina carolina*) and spur-thighed tortoises (*Testudo graeca*). Higher albumin concentrations were reported in female Hermann’s tortoises (*Testudo hermanni*) and spur-thighed tortoises, as well as in male Roti-island snake-necked turtles (*Chelodinia mccordi*) and yellow pond turtles (*Mauremys mutica*).^16,17,42,62^ Globulin concentrations varied between sexes in Eastern box turtles, Blanding’s turtles (*Emydoidea blandingii*), Hermann’s tortoises, painted turtles (*Chrysemys picta*), spur-thighed tortoises, Roti-island snake-necked turtles, Chinese stripe-necked turtles (*Ocadia sinensis*), and yellow pond turtles.^6,16,17,42,63,62,109^ Relative and absolute globulin concentrations varied between juveniles and adult Blanding’s turtles and gopher tortoises, as well as in gravid females.^6,17,94^ HBP was detected in higher concentrations in female and adult eastern box turtles.^42^

Given their ectothermic nature, turtle APP concentrations are also impacted by external factors such as temperature, geographic location, climate, exposure to contaminants or chemical toxins, habitat disturbance, and season.^6,25,41,50,56,77,86,113^ For example, plasma total protein and globulin concentrations in loggerhead sea turtles were higher in warmer water; significant negative relationships were noted between blood selenium, lead, cadmium, and arsenic concentrations and total protein, albumin, and globulin concentrations in loggerhead sea turtles.^79,89^ In particular, γ-globulins were reduced in the latter study, suggesting potential immune suppression in turtles as a result of exposure to toxic heavy metals in the environment.^89^

Nevertheless, increasing numbers of studies have established PEP RIs in some species and investigated the influence of these factors on protein fractions.

### Albumin

Albumin is a major negative APP in all species; the albumin concentration in blood decreases during acute inflammation. Albumin is a small protein that constitutes a large portion of the serum (35–50%), playing a major role in maintaining colloid osmotic pressure, blood volume, and as a transport protein.^34^ In turtles, blood albumin concentrations are decreased and are significantly lower in unhealthy animals or during inflammation, making a decreased albumin concentration a reliable marker of disease.^17,30,74,79,100^ Decreases in quantity are detected by both the BCG and PEP methods. However, the BCG method has reported higher than actual values of albumin in diseased animals compared with electrophoresis methods,^66,76^ and therefore should not be used to measure albumin concentrations in diseased turtles. Reduced albumin concentrations can result from loss associated with the APR, protein-losing diseases, and decreased hepatic synthesis.^34^

### Hemoglobin-binding protein, haptoglobin

Hp is a glycoprotein that binds free Hb in the blood. Free Hb has peroxidase activity that can cause oxidative injury to tissue; Hp binding prevents further tissue damage. By binding to Hb, Hp reduces the availability of heme residue and its iron on the Hb molecule for bacterial use, and hence has bacteriostatic activities.^13,34^ Hp also exerts anti-inflammatory effects by modulating the neutrophil respiratory burst, release of anti-inflammatory mediators, and suppression of T-cell proliferation.^13^ Reptiles lack the gene for Hp, but a HBP similar to the β-chain of mammalian Hp was detected in the Chinese soft-shelled turtle (*Trionyx sinensis*).^112^ The commercial colorimetric assay (Phase Hp assay; Tri-Delta Diagnostics) was validated and used in studies of box turtles and loggerhead sea turtles. HBP was found to be elevated in box turtles with clinical signs of disease or active injuries and elevated in loggerhead sea turtles during recovery from disease,^2,30,42^ suggesting its potential as an inflammation marker or prognostic indicator.

### Serum amyloid A (SAA)

SAA proteins are a family of small, highly conserved proteins in vertebrates, and a major APP in many mammals, where their concentration increases significantly in the face of an inflammatory insult.^35^ SAA is a small hydrophobic apolipoprotein closely associated with high-density lipoprotein. Excessive and persistently high SAA concentrations during chronic inflammatory conditions have been implicated with the pathologic deposition of amyloid A fibril, causing secondary amyloidosis.^96^ The biology of SAA remains unknown, but SAA is suggested to be involved in multiple pathways during acute inflammation, including the transport and recycling of cholesterol from sites of damaged tissue back to the liver.^34^ SAA also mediates the migration, adhesion, and tissue infiltration of monocytes and neutrophils, can opsonize bacteria, and subsequently increase leukocyte phagocytic activity.^13^ SAA is used frequently to provide insights into patient response to therapy and disease progression in some mammals.^108^

In turtles, elevated amounts of SAA mRNA were detected in the organs of Chinese soft-shelled turtles infected with *Aeromonas hydrophila*.^116,118^ However, in mammalian studies, elevated SAA mRNA amounts do not equate to elevated translated proteins, because post-transcription regulation plays a role in controlling blood SAA concentrations.^96,107^ Only one study has examined the clinical usefulness of blood SAA as a biomarker in sea turtle health. Plasma SAA concentrations were significantly higher in moribund loggerhead sea turtles than in recovered turtles, but also had a high interquartile range within the moribund group, and were not significantly correlated with clinical measurands in blood analyses.^72^ Samples from this study were also analyzed retrospectively from a small number (*n* = 15) of banked plasma, using an SAA assay specific for chicken SAA (Chicken SAA SPARCL assay; Life Diagnostics), with partial validation performed (linearity); results should therefore be interpreted as preliminary. The utility of SAA as an inflammation biomarker in reptiles therefore remains unknown and requires further research into its clinical application.

### Fibrinogen

Fibrinogen is a highly conserved APP in vertebrates and a minor-to-moderate positive APP in many species, constituting the largest proportion of plasma protein produced during the APR.^31^ Fibrinogen is a large protein made of 3 pairs of polypeptide chains, α2, β2, and γ2, in which the α- and β-chains can have great diversity among species.^31^ Fibrinogen serves as the precursor to fibrin, the key component of the coagulation cascade, and dysregulation is observed in disseminated intravascular coagulation.

mRNA expression of fibrinogen increased in Chinese soft-shelled turtles infected with *Aeromonas hydrophila*.^118^ However, in a study utilizing a modified Jacobsson method to quantify fibrinogen in red-eared sliders, no significant increase of the protein occurred in turtles infected with ranavirus.^75^ Fibrinogen has also been studied in box turtles and gopher tortoises.^82,93,94^ However, some of these studies lacked analytical or diagnostic performance validation and employed different measurement methods (modified Clauss or heat precipitation). Human-based assays may not reliably quantify fibrinogen and its different forms, such as fibrinogen α-chain, making their use as inflammation biomarkers inconclusive.^75,94^ Further research is needed to determine the clinical applicability of fibrinogen in turtles.

### Myeloid-related protein

MRP-126 expression in reptiles has been described, and studies have been undertaken to examine its potential as a biomarker in reptiles.^29,71,72^ MRP-126 is a member of the S100 protein family and a calgranulin homologue to mammalian calgranulin S100A12, which binds to mammalian toll-like receptor 4.^11,65^ MRP-126 is secreted by granulocytes, monocytes, early macrophages, keratinocytes, and endothelial and epithelial cells during inflammation.^44,114^ Its function includes inhibiting the growth of bacteria by binding zinc ions via a calcium-dependent action, promoting neutrophil adhesion to fibrinogen, and facilitating tissue repair.^11,104^ Although a commercial assay (Life Diagnostics) is available, only 2 studies have investigated the clinical usefulness of MRP-126 as an APP and inflammation marker. MRP-126 was elevated in 2 of 3 diseased Aldabra giant tortoises, but was also elevated in 3 of 27 healthy animals.^29^ In a study of banked plasma from loggerhead, Kemp’s ridleys (*Lepidochelys kempii*), and green turtles, plasma MRP-126 concentrations were significantly higher in moribund animals compared with recovered and healthy animals, although this had no significant association with disease etiology or turtle size.^72^ Clinically, MRP-126 concentrations also decreased as turtles recovered during rehabilitation. Despite the limitations of being a retrospective study with a small sample size, the same study concluded that MRP-126 was a reliable biomarker for sea turtles, offering a more accurate tool to categorize health status than other clinical measurands (such as total solids and WBC count), and a useful tool for monitoring recovery from general disease conditions. Until complete validation of the Life Diagnostics MRP-126 assay is reported, conclusions from current studies should be taken as preliminary, and additional research is required to determine if MRP-126 is useful in other turtle species.

### C-reactive protein (CRP)

CRP is a short pentraxin APP molecule that binds to C-polysaccharide of gram-positive bacteria, fungi, and parasites.^111^ Its main role in innate immunity includes activating and regulating the classical complement pathway and subsequent opsonization of bacteria, increasing anti-inflammatory cytokine production, modulating neutrophil function, and participating in tissue remodeling and repair to restore homeostasis.^35,111^ CRP is highly conserved in vertebrates, is a major APP in humans and dogs, and serves as an important APP for monitoring disease in pigs and mice. Although a universal CRP gene was reported in all vertebrates, no studies are available on CRP detection in reptiles despite being reported extensively in fish.^85,111^ Commercial assays for non-human primates and domestic animals are available but not for turtles. A pentraxin fusion protein-like molecule was found in higher abundance in green turtles exposed to organohalogen contaminants.^15^ Further studies are required to determine the presence, structure, and function of pentraxin-like molecules in turtle immunity.

### α2 macroglobulin (α2M)

α2M is a highly conserved and abundant protein in vertebrate plasma.^7^ Its main function in the innate immune system is to bind and neutralize a broad range of proteases released by parasites and microbes, as well as by phagocytes and other immune cells, thereby minimizing damage to tissue caused by protease enzymes during inflammation.^35^ α2M also plays an important role as a transmembrane cell receptor, transporting lipids and hormones, delivering antigen to antigen-presenting cells, and contributing to the inflammatory and subsequent homeostatic response of healing.^121^ α2M is a major APP in rats and a minor-to-moderate APP in humans.^18^

A tetrameric α2M homologous to human α2M was detected in green turtles.^78^ The abundance of α2M and its different forms increased or decreased in proteomic studies of plasma from different populations of sea turtles exposed to various concentrations of organohalogen contamination and in sea turtles undergoing rehabilitation and recovery, suggesting its potential as an inflammation biomarker.^15,54^ Unfortunately, although commercial ELISA kits are available for non-human primates and domestic animals, including mice, rats, cattle, equids, pigs, dogs, rabbits, and chickens, no kit is available for turtles.

## Conclusions

Since the early 2000s, APP studies in turtles have increased, enhancing our understanding of their potential as diagnostic support tools. Although PEP is highly sensitive in detecting disease or inflammation, similar to findings in mammals, PEP suffers from low specificity.^19,36,91,97^ Moreover, in reptiles, inflammation is not always corelated with conventional clinical findings, such as behavioral fever, leukocytosis, left shift, neutrophilia, or toxic changes,^17,50,72^ making APPs a valuable diagnostic support tool, while requiring further studies to expand the use of this modality.

However, significant challenges hinder the broader application of APP in turtles. Chief among these is the paucity of PEP RIs for healthy individuals, especially in exotic or endangered species. This gap stems from difficulties in recruiting study participants, limited access to specialized equipment or laboratories, and the logistical constraints of working with non-domesticated animals. Increasing accessibility to APP testing and emphasizing its importance to owners and clinicians is crucial. Another challenge is the scarcity of APP assays applicable in turtles given the lack of commercial species-specific reagents and control materials.

APP application is further complicated by variations in clinical practice and differing sample collection protocols, clinical measurands, and physiologic responses within species, influenced by factors such as sex, age, reproductive status, season, and environmental conditions.^119^ Further research should analyze these factors, and conventional clinical pathology, including blood counts and imaging studies, should be performed. Standardized sample collection protocols for serum or plasma and reporting of protein fractions in electrophoresis, to include quantitative and percentage values, are also necessary.

Compounding these issues is the high biological variability within and among turtle species. This variability complicates the interpretation of APP results and could potentially limit the utility of population-based RIs, which may fail to detect disease in individuals.^1^ Subject-based RIs, established through serial sampling of healthy animals, offer a promising alternative, especially for species without established RIs or for small, managed captive populations in conservation programs.^45^

To advance APP testing options for turtles, future research must prioritize biomarker identification, assay development and validation, standardized collection protocols, and comprehensive reporting of electrophoresis data. From a practical perspective, clinicians and researchers investigating disease or inflammation in turtles should include PEP in routine testing, setting up baseline RIs initially where feasible. Otherwise, trending of the electrophoretogram during disease or recovery progression in an individual is still useful. As MS-based proteomics become more affordable and accessible, concurrent species-specific genomic or transcriptomic sequencing must be performed to build the necessary library databases for bioinformatics analysis. Commercial availability of genomic and transcriptomic sequencing has increased rapidly in the past decade, while the cost has reduced and may be more easily accessible to researchers. Collaboration among laboratories, veterinary practices, and the research community is especially critical to harmonize methodologies and generate robust RIs. Ultimately, addressing the challenges posed by biological variation and limited data is essential for improving health monitoring and veterinary care in this diverse and ecologically important taxon.

## Supplemental Material

sj-pdf-1-vdi-10.1177_10406387261445937 – Supplemental material for Between the shells: a review of acute-phase proteins in turtles Supplemental material, sj-pdf-1-vdi-10.1177_10406387261445937 for Between the shells: a review of acute-phase proteins in turtles by Shin Min Chong, Carolyn Cray, Gabriele Rossi, Shangzhe Xie and Gordon S. Howarth in Journal of Veterinary Diagnostic Investigation
