# Supplementary material for: Between the shells: a review of acute-phase proteins in turtles
Source: J Vet Diagn Invest. 2026 Jun 6:10406387261445937. Online ahead of print. doi: 10.1177/10406387261445937 (PMC13242370; doi:10.1177/10406387261445937)
Supplement: sj-pdf-1-vdi-10.1177_10406387261445937 – Supplemental material for Between the shells: a review of acute-phase proteins in turtles [file sj-pdf-1-vdi-10.1177_10406387261445937.pdf]

Chong SM, et al. Between the shells: a review of acute-phase proteins in turtles

Supplemental Table 1. Acute-phase proteins and protein electrophoresis fractions influenced by biological or external factors.

		Factors influencing analytes and findings					
Species	Analyte	Sex	Age	Season	Reproductive status	Health status, presence of disease	Ref.
Eastern box turtle (*Terrapene carolina carolina*)	HBP, mg/mL	Yes; higher in females	Yes; higher in adults	Unknown	Unknown	NA	42
	TP, g/dL	Yes; higher in females	No	Yes; higher in summer	Unknown	NA	
	Pre-albumin, g/dL	No	No	No	Unknown	NA	
	Albumin, g/dL	No	No	Yes; higher in summer	Unknown	NA	
	α1, g/dL	Yes; higher in females	No	No	Unknown	NA	
	α2, g/dL	Yes; higher in females	No	No	Unknown	NA	
	β, g/dL	Yes; higher in females	Yes; higher in adults	No	Unknown	NA	
	γ, g/dL	Yes; higher in females	No	No	Unknown	NA	
	A:G ratio	No	No	No	Unknown	NA	
Blanding's turtles (*Emydoidea blandingii*)	TP, g/L	No	No	Yes; by year	No	Yes; higher in unhealthy	6
	Pre-albumin, g/L	No	No	Yes; by year & month	No	Yes; lower in unhealthy	
	Albumin, g/L	No	No	Yes; by year & month	Yes; gravid females lower	Yes; higher or lower in unhealthy	
	α1, g/L	No	Yes; juveniles highest	Yes; by year	No	No	
	α2, g/L	No	Yes; subadults highest	Yes; by year	Yes; gravid females lower	No	

	Analyte	Factors influencing analytes and findings: Sex	Age	Season	Reproductive status	Health status, presence of disease	Ref.
	β, g/L	Yes; females higher	Yes; adults highest	Yes; by year	No	Yes; higher or lower in unhealthy	
	γ, g/L	No	Yes; adults highest	Yes; by year	No	Yes; higher in unhealthy	
	A:G ratio	Yes; males higher	Yes; juveniles highest	Yes; by year & month	Yes; gravid females lower	Yes; lower in unhealthy	
	Pre-albumin, %	No	No	Yes; by year	No	Yes; lower in unhealthy	
	Albumin, %	Yes; males higher	Yes; juveniles highest	Yes; by year & month	Yes; gravid females lower	No	
	α1, %	No	Yes; juveniles highest	Yes; by year	No	No	
	α2, %	Yes; males higher	Yes; subadults highest	No	Yes; gravid females lower	No	
	β, %	Yes; females higher	Yes; adults highest	Yes; by year	Yes; gravid females higher	No	
	γ, %	No	Yes; adults highest	Yes; by year & month	No	No	
Radiated tortoise (*Geochelone radiata*)	TP, g/dL	No	Unknown	Yes; females, higher in summer	Unknown	NA	115
	Pre-albumin, g/dL	No	Unknown	Yes; males, higher in summer	Unknown	NA	
	Albumin, g/dL	No	Unknown	No	Unknown	NA	
	α1, g/dL	No	Unknown	Yes; females, higher in winter	Unknown	NA	
	α2, g/dL	No	Unknown	No	Unknown	NA	
	β, g/dL	No	Unknown	Yes; females, higher in winter	Unknown	NA	
	γ, g/dL	No	Unknown	No	Unknown	NA	
	A:G ratio	No	Unknown	No	Unknown	NA	
Hermann's tortoises (*Testudo hermanni*)	TP, g/dL	No	Unknown	Yes; lower in fall for males; lower in fall for females	Unknown	NA	62
	Albumin, g/dL	Yes; higher in females	Unknown	Yes; differs across seasons	Unknown	NA	

	Analyte	Factors influencing analytes and findings					Ref.
		Sex	Age	Season	Reproductive status	Health status, presence of disease	
	α, %	Yes; higher in males	Unknown	Yes; higher in fall for males; highest in fall for females	Unknown	NA	
	β, %	Yes; higher in males	Unknown	No	Unknown	NA	
	γ, %	No	Unknown	Yes; higher in spring for males; highest in fall for females	Unknown	NA	
	A:G ratio	Yes; higher in females	Unknown	Yes; higher in spring for males; highest in spring for females	Unknown	NA	
Gopher tortoises (*Gopherus polyphemus*)	ESR, mm/h	No	No	No	Unknown	NA	93
	Fibrinogen, mg/dL	No	No	No	Unknown	NA	
	TP, g/dL	No	Yes; immature higher	No	Unknown	NA	
	Pre-albumin, g/dL	No	Yes; immature higher	No	Unknown	NA	
	Albumin, g/dL	No	Yes; immature higher	No	Unknown	NA	
	α1, g/dL	No	Yes; immature higher	No	Unknown	NA	
	α2, g/dL	No	No	No	Unknown	NA	
	β, g/dL	No	No	No	Unknown	NA	
	γ, g/dL	No	No	No	Unknown	NA	
	A:G ratio	No	Yes; immature higher	No	Unknown	NA	
Painted turtles (*Chrysemys picta*)	TP, g/L	No	Unknown	NA	Unknown	No	109
	Pre-albumin, g/L	Yes; higher in females	Unknown	NA	Unknown	No	
	Albumin, g/L	No	Unknown	NA	Unknown	No	
	α1, g/L	No	Unknown	NA	Unknown	No	
	α2, g/L	No	Unknown	NA	Unknown	No	
	β, g/L	No	Unknown	NA	Unknown	No	
	γ, g/L	No	Unknown	NA	Unknown	No	
	A:G ratio	No	Unknown	NA	Unknown	No	

Species	Analyte	Factors influencing analytes and findings					Ref.
		Sex	Age	Season	Reproductive status	Health status, presence of disease	
Spur-thighed tortoise (*Testudo graeca*)	TP, g/L	Yes; higher in females	Unknown	NA (only fall)	Unknown	NA	63
	Albumin, g/L	Yes; higher in females	Unknown	NA (only fall)	Unknown	NA	
	Albumin, %	Yes; higher in females	Unknown	NA (only fall)	Unknown	NA	
	α, %	Yes; higher in males	Unknown	NA (only fall)	Unknown	NA	
	β, %	No	Unknown	NA (only fall)	Unknown	NA	
	γ, %	Yes; higher in males	Unknown	NA (only fall)	Unknown	NA	
Roti-island snake necked turtle (*Chelodina mccordi*)	TP, g/L	No	NA	No	Yes; lower in gravid females	Yes; lower in disease (*n* = 1)	17
	Pre-albumin, g/L	Yes; higher in males	NA	No	Yes; lower in gravid females	Yes; lower in disease (*n* = 1)	
	Albumin, g/L	Yes; higher in males	NA	No	Yes; lower in gravid females	Yes; lower in disease (*n* = 1)	
	α1, g/L	Yes; higher in females	NA	No	No	Yes; lower in disease (*n* = 1)	
	α2, g/L	No	NA	No	No	Yes; lower in disease (*n* = 1)	
	β, g/L	No	NA	No	No	Yes; lower in disease (*n* = 1)	
	γ, g/L	Yes; higher in females	NA	No	No	Yes; lower in disease (*n* = 1)	
	A:G ratio	Yes; higher in males	NA	No	Yes; lower in gravid females	No	
Aldabra giant tortoises (*Aldabrachlelys gigantea*)	TP, g/dL	Unknown	No	Unknown	Unknown	NA	29
	Pre-albumin 1, g/dL	Unknown	No	Unknown	Unknown	NA	
	Pre-albumin 2, g/dL	Unknown	No	Unknown	Unknown	NA	
	Albumin, g/dL	Unknown	No	Unknown	Unknown	NA	
	α1, g/dL	Unknown	No	Unknown	Unknown	NA	
	α2, g/dL	Unknown	No	Unknown	Unknown	NA	
	β, g/dL	Unknown	No	Unknown	Unknown	NA	
	γ, g/dL	Unknown	No	Unknown	Unknown	NA	

Species	Analyte	Factors influencing analytes and findings					Ref.
		Sex	Age	Season	Reproductive status	Health status, presence of disease	
	A:G ratio	Unknown	No	Unknown	Unknown	NA	
	MRP-126, mg/L	Unknown	No	Unknown	Unknown	NA	
Chinese stripe-necked turtle (*Ocadia sinensis*)	TP, g/dL	Unknown	Unknown	No	NA	NA	16
	Albumin, g/dL	No	Unknown	No	NA	NA	
	Albumin, %	Yes; higher in males	Unknown	No	NA	NA	
	α, g/dL	Yes; higher in females	Unknown	Yes; higher in summer	NA	NA	
	α, %	No	Unknown	Yes; higher in summer	NA	NA	
	β, g/dL	No	Unknown	Yes; lower in winter	NA	NA	
	β, %	No	Unknown	No	NA	NA	
	γ, g/dL	No	Unknown	No	NA	NA	
	γ, %	No	Unknown	Yes; higher in summer	NA	NA	
	A:G ratio	Yes; higher in males	Unknown	No	NA	NA	
Yellow pond turtle (*Mauremys mutica*)	TP, g/dL	Unknown	Unknown	No	NA	NA	16
	Albumin, g/dL	Yes; higher in males	Unknown	Yes; higher in spring	NA	NA	
	Albumin, %	No	Unknown	Yes; higher in winter	NA	NA	
	α1, g/dL	No	Unknown	No	NA	NA	
	α1, %	Yes; higher in females	Unknown	Yes; higher in spring	NA	NA	
	α2, g/dL	No	Unknown	No	NA	NA	
	α2, %	Yes; higher in females	Unknown	Yes; higher in spring	NA	NA	
	β, g/dL	Yes; higher in males	Unknown	Yes; higher in spring	NA	NA	
	β, %	No	Unknown	Yes; higher in autumn	NA	NA	
	γ, g/dL	Yes; higher in males	Unknown	Yes; higher in spring	NA	NA	
	γ, %	Yes; higher in males	Unknown	Yes; higher in summer	NA	NA	
	A:G ratio	No	Unknown	Yes; higher in winter	NA	NA	

Species	Analyte	Factors influencing analytes and findings: Sex	Age	Season	Reproductive status	Health status, presence of disease	Ref.
Spotted turtles (*Clemmys guttata*)	TP, g/dL	Unknown	NA	NA	Unknown	NA	12
	Pre-albumin, g/dL	Unknown	NA	NA	Unknown	NA	
	Albumin, g/dL	Unknown	NA	NA	Unknown	NA	
	α1, g/dL	Unknown	NA	NA	Unknown	NA	
	α2, g/dL	Unknown	NA	NA	Unknown	NA	
	β1, g/dL	Unknown	NA	NA	Unknown	NA	
	β2, g/dL	Unknown	NA	NA	Unknown	NA	
	Total β, g/dL	Unknown	NA	NA	Unknown	NA	
	γ, g/dL	Unknown	NA	NA	Unknown	NA	
	A:G ratio	Unknown	NA	NA	Unknown	NA	
Red-eared slider (*Trachemys scripta*)	TP, g/L	NA; all females	Unknown	NA; all sampled in spring	Unknown	NA	50
	Albumin, g/L	NA; all females	Unknown	NA; all sampled in spring	Unknown	NA	
	α, g/L	NA; all females	Unknown	NA; all sampled in spring	Unknown	NA	
	β, g/L	NA; all females	Unknown	NA; all sampled in spring	Unknown	NA	
	γ, g/L	NA; all females	Unknown	NA; all sampled in spring	Unknown	NA	
	A:G ratio	NA; all females	Unknown	NA; all sampled in spring	Unknown	NA	

Species	Location	Analyte	Factors influencing analytes and findings: Sex	Age/size	Season	Reproduction status	Health status/presence of disease	Ref.
Green turtle (*Chelonia mydas*)	Queensland, Australia	TP, g/L	Unknown	No	Unknown	Unknown	NA	40
		Pre-albumin, g/L	Unknown	Yes	Unknown	Unknown	No	
		Albumin, g/L	Unknown	No	Unknown	Unknown	Yes; elevated in foreign body cases	
		α, g/L	Unknown	No	Unknown	Unknown	Yes; elevated in unhealthy	

	Location	Analyte	Factors influencing analytes and findings: Sex	Age/size	Season	Reproduction status	Health status/presence of disease	Ref.
		β, g/L	Unknown	No	Unknown	Unknown	No	
		γ, g/L	Unknown	No	Unknown	Unknown	Yes; elevated in disease	
		β - γ, g/L	Unknown	No	Unknown	Unknown	No	
	Florida, USA	TP, g/L	NA	NA; all juveniles	Yes; higher in warmer water	NA	Unknown	79
		Albumin, g/L	NA	NA; all juveniles	No	NA	Unknown	
		α, g/L	NA	NA; all juveniles	Yes; higher in warmer water	NA	Unknown	
		β, g/L	NA	NA; all juveniles	No	NA	Unknown	
		γ, g/L	NA	NA; all juveniles	No	NA	Unknown	
		A:G ratio	NA	NA; all juveniles	Unknown	NA	Unknown	
Loggerhead turtle (*Caretta caretta*)	Georgia, USA	TP, g/L	No	NA	Unknown	Yes; highest in nesting	Yes; highest in nesting	27
		Pre-albumin, g/L	No	NA	Unknown	No	No	
		Albumin, g/L	No	NA	Unknown	Yes; highest in nesting	No	
		α1, g/L	No	NA	Unknown	No	No	
		α2, g/L	No	NA	Unknown	No	No	
		β, g/L	No	NA	Unknown	No	No	
		γ, g/L	No	NA	Unknown	No	No	
	Florida, USA	TP, g/dL	NA	No	Yes; higher in warmer water	NA	Yes; lower in disease	79
		Albumin, g/dL	NA	Yes; lower in smaller	No	NA	Yes; lower in disease	
		α, g/dL	NA	No	Yes; higher in warmer water	NA	Yes; lower in disease	
		β, g/dL	NA	No	Yes; higher in warmer water	NA	Yes; lower in disease	
		γ, g/dL	NA	Yes; higher in larger	Yes; higher in warmer water	NA	No	

	Location	Analyte	Factors influencing analytes and findings: Sex	Age/size	Season	Reproduction status	Health status/presence of disease	Ref.
		A:G ratio	NA	No	Yes; lower in warmer water	NA	Yes; lower in disease	
	Georgia, USA	HBP, mg/mL	NA	NA	NA	NA	Yes; increased during recovery	30
		TP, g/dL	NA	NA	NA	NA	Yes; increased during recovery	
		Albumin, g/dL	NA	NA	NA	NA	Yes; increased during recovery	
		α, g/dL	NA	NA	NA	NA	Yes; increased during recovery	
		β, g/dL	NA	NA	NA	NA	Yes; increased during recovery	
		γ, g/dL	NA	NA	NA	NA	Yes; increased during recovery	
Leatherback turtle (*Dermochelys coriacea*)	Republic of Gabon coast	TP, g/L	NA; all females	NA	Unknown	NA	NA	26
		Pre-albumin, g/L	NA; all females	NA	Unknown	NA	NA	
		Albumin, g/L	NA; all females	NA	Unknown	NA	NA	
		α1, g/L	NA; all females	NA	Unknown	NA	NA	
		α2, g/L	NA; all females	NA	Unknown	NA	NA	
		β, g/L	NA; all females	NA	Unknown	NA	NA	
		γ, g/L	NA; all females	NA	Unknown	NA	NA	
	US Virgin Islands	TP, g/dL	NA; all females	NA	Yes; decreased over season	NA; all nesting	Unknown	87
		Albumin, g/dL	NA; all females	NA	Yes; decreased over season	NA; all nesting	Unknown	
		α1, g/dL	NA; all females	NA	No	NA; all nesting	Unknown	
		α2, g/dL	NA; all females	NA	No	NA; all nesting	Unknown	
		β, g/dL	NA; all females	NA	Yes; decreased over season	NA; all nesting	Unknown	
		γ, g/dL	NA; all females	NA	Yes; decreased over season	NA; all nesting	Unknown	
		A:G ratio	NA; all females	NA	No	NA; all nesting	Unknown	
Kemp's ridley sea turtle	Georgia USA	TP, g/L	Unknown	Yes; positive association	NA	NA	NA	90

	Analyte	Factors influencing analytes and findings				
		Sex	Age/size	Season	Reproduction status	Health status/presence of disease
(*Lepidochelys kempii*)	Pre-albumin, g/L	Unknown	Yes; positive association	NA	NA	NA
	Albumin, g/L	Unknown	Yes; positive association	NA	NA	NA
	α1, g/L	Unknown	Yes; positive association	NA	NA	NA
	α2, g/L	Unknown	Yes; positive association	NA	NA	NA
	β, g/L	Unknown	Yes; positive association	NA	NA	NA
	γ, g/L	Unknown	Yes; positive association	NA	NA	NA
	A:G ratio	Unknown	No	NA	NA	NA

A:G ratio = albumin: globulin ratio, ESR = erythrocyte sedimentation rate, HBP = hemoglobin-binding protein; MRP-126 = myeloid-related protein 126, NA = not done or not applicable because only one variable used; TP = total protein; Unknown = variable/parameter/measurement present but not tested.
